# Multifactorial Determinants of Body Composition in the Korean Older Adults: Using Data from the 2022–2023 National Health and Nutrition Examination Survey

**DOI:** 10.3390/nu17091477

**Published:** 2025-04-27

**Authors:** Moonkyoung Park, ThiThu-Huyen Do, Jinsun Park

**Affiliations:** College of Nursing, Chungnam National University, Daejeon 35015, Republic of Korea; lunarnr@cnu.ac.kr (M.P.); dieuhuyen9122@gmail.com (T.-H.D.)

**Keywords:** sarcopenia, abdominal obesity, sarcopenic obesity, biopsychosocial model, older adults

## Abstract

**Background/Objectives**: Sarcopenia, abdominal obesity, and sarcopenic obesity are prevalent and clinically significant in older adults, each shaped by diverse biopsychosocial factors. However, integrative analyses using nationally representative data remain limited in Korea. **Methods**: We analyzed 2118 adults aged ≥65 years from the 2022–2023 Korea National Health and Nutrition Examination Survey (KNHANES). Body composition was classified into sarcopenia, abdominal obesity, and sarcopenic obesity. Guided by Engel’s Biopsychosocial Model, we examined biological (e.g., sex, chronic disease, nutrition, exercise), psychological (e.g., stress, sleep, self-rated health), and social (e.g., income, education, living status) variables. Complex-sample multinomial logistic regression identified condition-specific associations. **Results**: Prevalence rates were 18.2% for sarcopenia, 41.0% for abdominal obesity, and 3.4% for sarcopenic obesity. Eating alone and a lack of resistance exercise were common risk factors across all three conditions. Sarcopenia was associated with male sex, insufficient dietary intake, alcohol consumption, poor self-rated health, and low household income. Abdominal obesity was linked to recent weight gain, hypertension, diabetes, prolonged sedentary time, perceived obesity, and low educational attainment. Sarcopenic obesity was associated with male sex, diabetes, elevated hs-CRP, perceived stress, poor self-rated health, and economic inactivity. **Conclusions**: Body composition abnormalities among older Korean adults are influenced by complex, condition-specific interactions across biological, psychological, and social domains. These findings emphasize the significance of adopting an integrative perspective that considers physical, psychological, and social health components when addressing age-related body composition issues.

## 1. Introduction

The global increase in the aging population presents pressing public health challenges, particularly concerning abdominal obesity, sarcopenia, and sarcopenic obesity. The growing prevalence of these interconnected conditions among the elderly is linked to heightened risks of morbidity, functional decline, and mortality, placing considerable strain on individuals, caregivers, and healthcare infrastructures [1,2].

Abdominal obesity, characterized by excessive visceral fat accumulation, is closely linked to metabolic syndrome, cardiovascular disease, and all-cause mortality [1]. Age-related sarcopenia—marked by reductions in skeletal muscle mass and function—intensifies physical impairment and is strongly linked to increased incidences of falls, fractures, mobility challenges, and elevated healthcare use [2]. When these conditions coexist, they manifest as sarcopenic obesity, a clinical phenotype marked by excess fat and reduced muscle mass. This dual burden is associated with heightened risks of frailty, cardiometabolic diseases, physical dysfunction, and diminished quality of life [3,4]. As the global elderly population expands, the burden of sarcopenia and sarcopenic obesity is expected to grow.

Although previous studies have identified various risk factors—such as inadequate protein intake [5], smoking [6], low physical activity [7], metabolic disturbances, and psychological factors like depression [8,9]—most previous research has examined these domains in isolation. As such, a holistic understanding of how these factors interact remains limited. Additionally, emerging evidence suggests that lifestyle patterns and dietary behaviors established in early adulthood may influence the development of body composition abnormalities later in life. For instance, a study among young adults in Southern Italy demonstrated that nutritional education significantly improved health-related behaviors, highlighting the importance of preventive strategies across the lifespan [10].

In Korea, where population aging is among the most rapid worldwide, recent studies report prevalence rates of 24.5% for abdominal obesity, 27.4% for sarcopenia, and 4.4% for sarcopenic obesity among older adults [11,12]. Cultural dietary practices, sociodemographic patterns, and mental health characteristics unique to Korea necessitate a comprehensive, context-sensitive approach. While previous Korean and international studies have largely focused on biomedical, nutritional, or behavioral factors in isolation, few have adopted a comprehensive framework that captures the complex interplay between biological, psychological, and social domains.

Although prior studies have examined individual or dual aspects of the biopsychosocial framework (e.g., biological and psychological, or psychological and social), an integrative approach simultaneously encompassing all three domains remains relatively underexplored [13,14,15].

**Figure 1:**
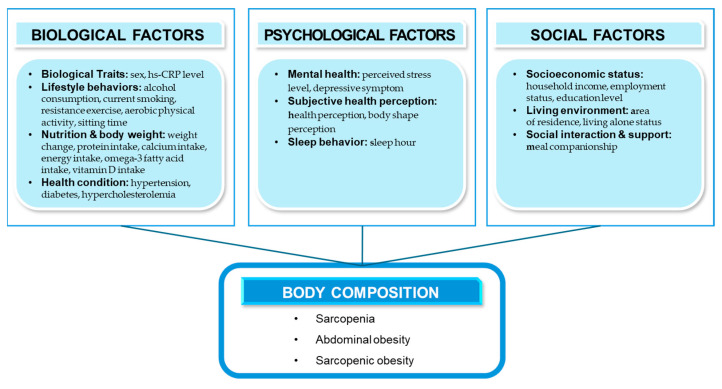
Conceptual model of body composition abnormalities grounded in Engel’s Biopsychosocial Theory.

To address this gap, the present study applies Engel’s Biopsychosocial Model [16], which captures the complex interplay of biological, psychological, and social determinants of health. As shown in Figure 1, The biological domain includes sex, smoking, alcohol consumption, nutritional intake (protein, calcium, energy, omega-3, and vitamin D), chronic conditions (hypertension, diabetes, hypercholesterolemia), weight change, hs-CRP levels, and behavioral factors (resistance and aerobic exercise, and sedentary behavior). The psychological domain includes perceived stress, depression, sleep duration, self-rated health, and body image. The social domain encompasses residence, income, employment, education, living arrangements, and whether meals are taken alone.

Using 2022–2023 data from the Korea National Health and Nutrition Examination Survey (KNHANES) [17], this study applies a holistic, multidimensional model to identify interrelated biopsychosocial determinants of abdominal obesity, sarcopenia, and sarcopenic obesity in Korean adults aged 65 and older. The findings aim to guide the development of tailored, person-centered public health interventions that address the complex needs of Korea’s rapidly aging population.

## 2. Materials and Methods

### 2.1. Research Design and Sample

This study is an observational cross-sectional analysis, conducted as a secondary analysis in accordance with the official guidelines for analyzing data from the 2022 and 2023 KNHANES [17]. KNHANES is a government-led, nationally representative survey administered annually using a rolling, multistage stratified cluster sampling design based on the Population and Housing Census. The dataset used in this study corresponds to the first and second years of the ninth KNHANES cycle.

Participants completed structured online health questionnaires, physical examinations, and dietary assessments using validated instruments and protocols. All household members aged one year or older were eligible to participate. A total of 6265 individuals participated in 2022, and 6929 individuals in 2023, across 192 survey clusters nationwide.

For this study, we analyzed data from 2118 community-dwelling older adults aged 65 years and above who had complete information on anthropometric, biochemical, lifestyle, psychological, and sociodemographic variables. Participants were classified into four groups based on body composition abnormalities using established diagnostic criteria [18]: normal (n = 776), sarcopenia (n = 392), abdominal obesity (n = 875), and sarcopenic obesity (n = 75).

According to a global meta-analysis by Gao and colleagues [19], the prevalence of sarcopenic obesity among older adults is approximately 11%. Using this estimate, and applying a relaxed threshold of 5 events per variable (EPV = 5) as recommended by Vittinghoff and McCulloch [20], the minimum sample size required to detect 135 events in a model including 27 covariates is 1227 participants under a simple random sampling assumption. After accounting for the complex survey design (design effect = 1.5), the adjusted minimum required sample size is approximately 1841 participants. As our final analytic sample consisted of 2118 individuals, the sample was deemed sufficient to yield stable estimates in multivariable logistic regression analyses.

### 2.2. Measurements

The variables used to assess biological, psychological, and social factors are summarized in Table 1. The diagnostic criteria for sarcopenia were based on cut-off values specific to Asian populations [18], while abdominal obesity was defined according to national standards for Korean adults [21]. In this study, sarcopenia was classified as low skeletal muscle mass in the absence of abdominal obesity, whereas abdominal obesity was defined as excess abdominal fat without concurrent sarcopenia. Sarcopenic obesity referred to the coexistence of both conditions—low muscle mass and abdominal obesity.

### 2.3. Statistical Analysis

In this study, complex sample analyses were conducted using IBM SPSS Statistics (version 29.0; IBM Corp., Armonk, NY, USA), following the procedures recommended in the KNHANES Raw Data User Manual. Descriptive statistics, including frequencies and percentages, were used to examine the distribution of biological, psychological, and social factors across body composition abnormalities. Complex-sample logistic regression analyses were then performed to identify factors significantly associated with the presence of sarcopenia, abdominal obesity, and sarcopenic obesity.

## 3. Results

### 3.1. Prevalence and Characteristics of Sarcopenia, Obesity, and Sarcopenic Obesity

As shown in Table 2, among the 2118 older adults included in the analysis, 776 (37.4%) were classified as normal, 392 (18.2%) had sarcopenia, and 876 (41.0%) had abdominal obesity. Sarcopenic obesity was identified in 75 individuals (3.4%). The table also presents the distribution of biological, psychological, and social factors across the four body composition status groups. Notably, none of the participants in the sarcopenia group perceived themselves as obese, and no individuals in the sarcopenic obesity group reported sufficient vitamin D intake.

### 3.2. Associations of Biological, Psychological, and Social Factors with Body Composition Abnormalities in Older Adults

**Figure 2:**
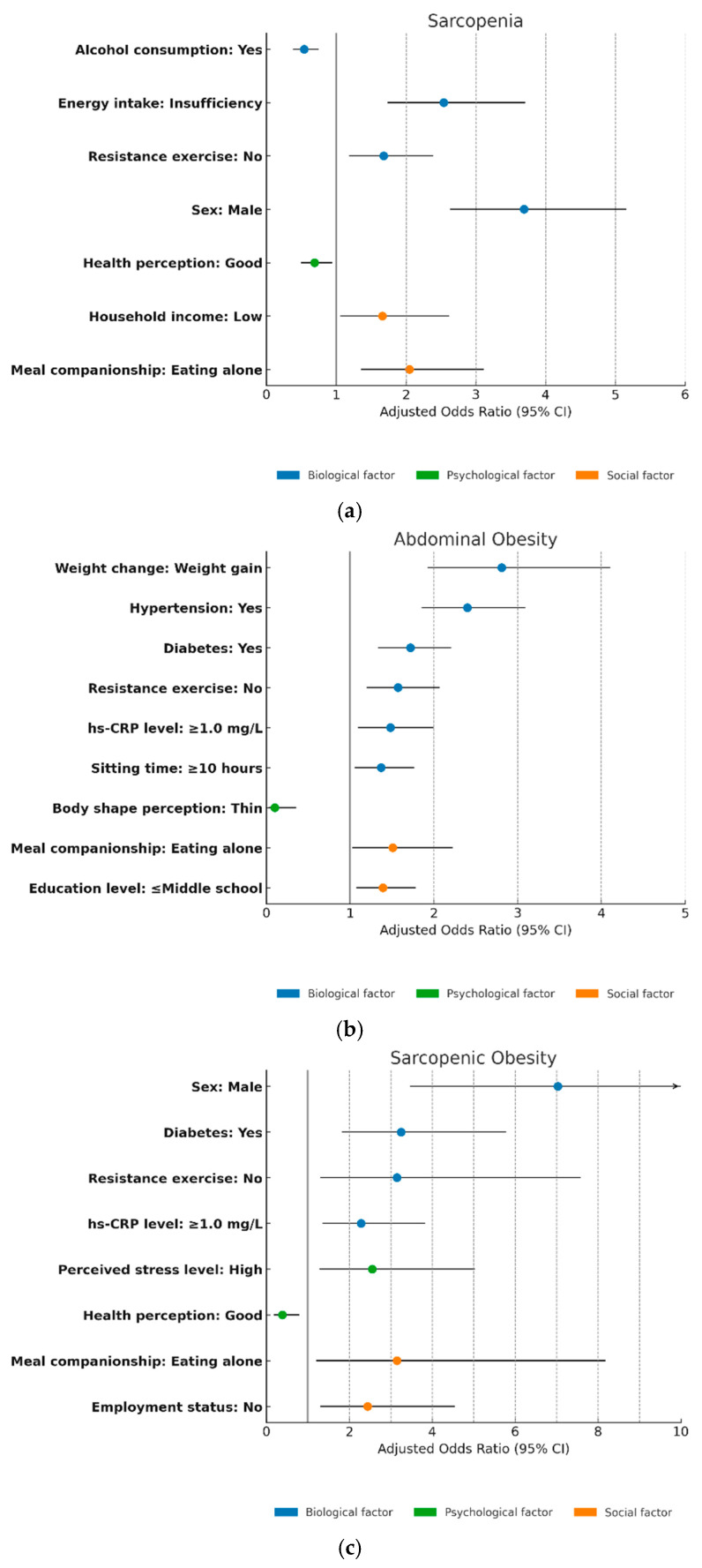
Forest plots of significant predictors of body composition abnormalities: (**a**) sarcopenia group; (**b**) abdominal obesity group (Note: Perceived obesity [AOR = 50.99, 95% CI: 12.94–200.88] was excluded for visual clarity); (**c**) sarcopenic obesity group (The upper bound of the 95% CI for ‘Sex: Male’ exceeded the x-axis limit and is indicated with an arrow to denote continuation beyond the scale [AOR > 10]).

Table 3 and Figure 2 present the associations between categories of body composition abnormalities and biological, psychological, and social factors among older adults.

For sarcopenia, several variables showed significant associations. Among the biological factors, male sex (AOR = 3.69, 95% CI: 2.64–5.15), insufficient dietary intake (AOR = 2.54, 95% CI: 1.74–3.70), and a lack of resistance exercise (AOR = 1.68, 95% CI: 1.19–2.38) were positively associated with sarcopenia. In contrast, alcohol consumption was associated with a reduced risk (AOR = 0.54, 95% CI: 0.39–0.74). Among the psychological factors, perceiving oneself to be in good health was significantly associated with a reduced risk (AOR = 0.69, 95% CI: 0.50–0.94). Social factors such as eating alone and low household income were also linked to an increased risk of sarcopenia (AOR = 2.05, 95% CI: 1.36–3.11; AOR = 1.66, 95% CI: 1.06–2.61, respectively) (Figure 2a).

In the case of abdominal obesity, a range of biological, psychological, and social factors demonstrated significant associations. Among the biological factors, recent weight gain showed the strongest association (AOR = 2.81, 95% CI: 1.93–4.10), followed by hypertension (AOR = 2.40, 95% CI: 1.86–3.09), diabetes (AOR = 1.72, 95% CI: 1.34–2.20), a lack of resistance exercise (AOR = 1.57, 95% CI: 1.20–2.06), elevated hs-CRP levels (AOR = 1.48, 95% CI: 1.10–1.99), and prolonged sedentary behavior (AOR = 1.37, 95% CI: 1.06–1.76). Among psychological factors, perceiving oneself as obese was strongly associated with increased risk (AOR = 50.99, 95% CI: 12.94–200.88), while perceiving oneself as thin was linked to a decreased risk (AOR = 0.10, 95% CI: 0.03–0.35). Regarding social factors, eating alone (AOR = 1.51, 95% CI: 1.03–2.22) and low educational attainment (middle school or below) (AOR = 1.39, 95% CI: 1.08–1.78) were significantly associated with abdominal obesity (Figure 2b).

In the context of sarcopenic obesity, multiple biological, psychological, and social correlates were observed. Among the biological factors, male sex demonstrated the strongest association (AOR = 7.03, 95% CI: 3.48–14.20), followed by diabetes (AOR = 3.25, 95% CI: 1.83–5.77), insufficient resistance exercise (AOR = 3.15, 95% CI: 1.31–7.57), and elevated hs-CRP levels (AOR = 2.28, 95% CI: 1.36–3.81). Among psychological factors, high perceived stress was associated with an increased risk (AOR = 2.55, 95% CI: 1.29–5.01), whereas a good self-perceived health status was associated with a decreased risk (AOR = 0.38, 95% CI: 0.19–0.78). With respect to social factors, eating alone (AOR = 3.15, 95% CI: 1.21–8.17) and economic inactivity (AOR = 2.44, 95% CI: 1.32–4.53) were both positively associated with sarcopenic obesity (Figure 2c).

## 4. Discussion

This study examined the associations between biological, psychological, and social factors and the presence of sarcopenia, abdominal obesity, and sarcopenic obesity in Korean older adults, using Engel’s Biopsychosocial Model [16]. Leveraging nationally representative data from the 2022–2023 KNHANES [17], we adopted a multidimensional approach to better understand the complex determinants of body composition in aging populations. The prevalence of sarcopenia, abdominal obesity, and sarcopenic obesity was identified as 18.2%, 41.0%, and 3.4%, respectively. Our findings revealed that the factors influencing body composition abnormalities differed by condition, underscoring the importance of condition-specific strategies.

For sarcopenia, male sex, insufficient dietary intake, and a lack of resistance exercise were positively associated, in line with previous studies highlighting the role of nutritional deficiency and physical inactivity in muscle loss among older adults [5,6,7,8,9]. Interestingly, alcohol consumption was associated with a lower risk of sarcopenia. While the protective role of moderate alcohol consumption is debated, some research suggests that social drinking may correlate with higher physical activity or social engagement, potentially buffering muscle loss [6]. However, it may reflect confounding by social engagement or healthier lifestyle behaviors, though further research is needed.

Psychological and social variables also contributed to sarcopenia risk. Older adults who perceived their health as good were less likely to have sarcopenia, suggesting the protective role of positive self-rated health. In contrast, low household income and eating alone were associated with increased risk, reflecting the adverse influence of socioeconomic disadvantage and social isolation [6,12]. Interestingly, none of the participants in the sarcopenia group perceived themselves as obese, highlighting potential misperception of body image that may delay risk recognition or behavior change [34].

In the case of abdominal obesity, biological risk factors included recent weight gain, hypertension, diabetes, lack of resistance exercise, elevated hs-CRP, and prolonged sedentary behavior. These findings are consistent with prior research linking visceral adiposity to metabolic dysfunction and inflammation [1,12,18,21,35]. Structured resistance training, reducing sedentary time, and early weight management could therefore serve as key intervention targets.

Behavioral risk factors, particularly a lack of resistance exercise and sedentary behavior, are known to reduce fat oxidation and muscle activity, accelerating fat accumulation. Silveira and colleagues [36] found prolonged sitting to be positively associated with abdominal obesity, while Skrypnik and colleagues [37] demonstrated that structured exercise can significantly improve metabolic outcomes in individuals with visceral adiposity. Weight gain, particularly in the context of low energy expenditure, is also a key driver. Slentz and colleagues [38] noted that reduced fat oxidation during inactivity is predictive of central fat accumulation. Hypertension and vascular dysfunction may reduce muscle perfusion and oxygenation, as reported by De Lima and colleagues [39] and Ungvari and colleagues [40], thereby impairing muscle regeneration. Structured resistance training, reducing sedentary time, and early weight management could therefore serve as key intervention targets. These findings highlight the need for comprehensive behavioral interventions, including dietary improvement, increased physical activity, and reduced sedentary time.

Psychologically, self-perceived obesity was positively associated with abdominal obesity, while perceiving oneself as thin was associated with reduced risk. These findings suggest that body image perception may influence lifestyle engagement and weight management [12]. Socially, lower educational attainment and eating alone were significant contributors, indicating the importance of health literacy and supportive environments in managing abdominal obesity.

Sarcopenic obesity, characterized by the co-occurrence of low muscle mass and high fat mass, showed distinct risk patterns. Male sex, diabetes, insufficient resistance exercise, and elevated hs-CRP were all significantly associated, suggesting a combined effect of metabolic dysfunction and inflammation [2,3,4,18,21]. Elevated hs-CRP levels highlight the role of systemic inflammation, promoting muscle catabolism [3,5,30]. Cardiometabolic conditions further contribute via oxidative stress and insulin resistance [41,42]. Importantly, none of the participants in this group reported sufficient vitamin D intake, reinforcing the hypothesis that vitamin D deficiency may contribute to muscle-fat imbalance [43,44,45].

Resistance training has demonstrated efficacy in mitigating these effects by improving insulin sensitivity and promoting hypertrophy [46]. Khadra and colleagues [47] further reported a higher prevalence of metabolic syndrome in individuals with sarcopenic obesity compared to those with sarcopenia or obesity alone.

Psychologically, high perceived stress and poor self-rated health were linked to sarcopenic obesity, pointing to the influence of mental health. Stress-related hormonal changes and behavioral effects such as emotional eating may exacerbate both fat gain and muscle loss [4,6]. Social variables including economic inactivity and eating alone reinforce the significance of financial and social support systems [12,21]. These findings support the concept that sarcopenic obesity arises from cumulative biological, psychological, and social disadvantages.

These interrelated risks highlight the need for integrated health strategies. Individuals with limited income or social support may be less likely to exercise or maintain balanced diets, amplifying vulnerability. Community-level interventions—such as group meals, social prescribing, and structured physical activity programs—could address both social isolation and lifestyle behaviors. Social prescribing, in particular, offers promise by connecting older adults with non-medical community services to improve engagement and well-being [48,49].

The results highlight critical considerations for the development of evidence-based health policies targeting older adults. They support the development of multidimensional interventions that address not only physical and nutritional components but also psychosocial factors [16]. Screening for social isolation, subjective health perceptions, and dietary habits could help identify vulnerable older adults. Interventions promoting resistance training and communal meals may serve as effective, scalable measures for preventing sarcopenia and related conditions [12,18].

The observed associations between low socioeconomic status, living alone, and body composition abnormalities further underscore the need for community-level support systems, such as meal-sharing programs, age-friendly fitness initiatives, and targeted mental health services.

A notable strength of the present study lies in the utilization of nationally representative data, which strengthens the external validity of the results and supports their applicability to the wider older adult population in Korea [17]. The application of Engel’s Biopsychosocial Model enabled a nuanced understanding of multifactorial determinants of abnormal body composition [16].

Despite its contributions, this study has limitations. Its cross-sectional design constrains causal interpretation and prevents the determination of temporal order, raising concerns about reverse causality. For instance, poor physical function or depressive symptoms may not only be outcomes of body composition abnormalities but also antecedents, influencing behaviors such as reduced physical activity, inadequate nutrition, or social withdrawal. The reliance on self-reported measures (e.g., sleep, perceived health) may further introduce measurement bias. Additionally, interaction effects (e.g., between sex and income) were not examined and warrant further exploration. While extensive adjustments were made for potential confounders, residual confounding, and bias due to the exclusion of participants with incomplete data cannot be entirely ruled out. These factors may have affected the generalizability and precision of our findings. The absence of certain responses in specific subgroups (e.g., no sufficient vitamin D intake reported in the sarcopenic obesity group) may have resulted in separation issues in regression models, potentially affecting estimate reliability. Future longitudinal studies with objective measures and more robust adjustments for unmeasured confounders are warranted.

In conclusion, this study advances the existing literature by utilizing Engel’s Biopsychosocial Model to investigate atypical body composition patterns among older adults. Unlike previous studies that examined biological, psychological, or social domains in isolation, our approach underscores the interactive and cumulative nature of health determinants. For example, individuals experiencing economic inactivity may also suffer from nutritional inadequacy and psychological stress, while those who eat alone may lack not only social support but also motivation for physical activity. These overlapping disadvantages can synergistically increase vulnerability to sarcopenia, abdominal obesity, and sarcopenic obesity—risks that are often underestimated in unidimensional frameworks.

Our findings have important practical implications for public health and aging policy. Common risk factors such as social isolation and lack of resistance exercise highlight the need for multidimensional interventions that integrate physical, psychological, and social domains. Community-based strategies—such as meal-sharing initiatives, structured resistance training, peer support groups, and social prescribing—may be particularly effective in reducing health risks and enhancing overall well-being. Tailoring these approaches to the sociocultural context of Korea is essential for ensuring relevance, accessibility, and scalability.

Future research should explore these multidimensional interactions more explicitly using longitudinal designs, pathway analyses, or structural equation modeling. Such work would clarify causal relationships and inform the development of person-centered interventions that address the intertwined biological, psychological, and social challenges of aging. Expanding research on underexplored populations and incorporating culturally tailored approaches will further strengthen efforts to promote healthy aging in rapidly aging societies.

## 5. Conclusions

This study identified biological, psychological, and social factors associated with sarcopenia, abdominal obesity, and sarcopenic obesity in older Korean adults using data from the 2022–2023 KNHANES. The prevalence rates—18.2% for sarcopenia, 41.0% for abdominal obesity, and 3.4% for sarcopenic obesity—underscore the growing public health burden posed by these conditions in aging populations.

Common risk factors across the three conditions included eating alone and a lack of resistance exercise. Other key variables—such as male sex, inadequate nutritional intake, chronic disease, elevated hs-CRP, high perceived stress, and social isolation—also showed condition-specific associations. These findings emphasize the significance of adopting an integrative perspective that considers physical, psychological, and social health components when addressing age-related body composition issues.

Tailored strategies—such as promoting resistance training, ensuring balanced nutrition, and encouraging social engagement—may be particularly effective in mitigating health risks related to body composition abnormalities and enhancing the quality of life in older adults. Public health initiatives should prioritize integrated and culturally tailored approaches that address the complex and interconnected determinants of health in Korea’s rapidly aging society.

## Figures and Tables

**Table 1:** Description of biological, psychological, and social factors.

Variable	Variable Description
Body composition-based classification	
Sarcopenia	An SMI of less than 7.0 kg/m^2^ in men and less than 5.4 kg/m^2^ in women, in the absence of abdominal obesity, was used to define sarcopenia. SMI = ASM (kg)/height^2^ (m^2^) [22,23].
Abdominal obesity	In the absence of sarcopenia, abdominal obesity is characterized by a waist measurement of 90 cm or more in men and 85 cm or more in women [24].
Sarcopenic obesity	Defined as the coexistence of sarcopenia and abdominal obesity.
Biological factors	
Sex	Male, Female.
Alcohol consumption	Classified as “Yes” if consumed alcohol at least once a month in the past year; otherwise “No”.
Current smoking	Classified as “Yes” if smoked ≥5 packs lifetime and currently smoking; otherwise “No”.
Protein intake	Adequacy of daily protein intake: defined as “Sufficient” when ≥50 g for males and ≥45 g for females; otherwise, considered “Insufficient” [25].
Calcium intake	Adequacy of daily calcium intake: defined as “Sufficient” when ≥700 mg; otherwise, considered “Insufficient” [25].
Energy intake	Adequacy of daily energy intake recommendations for Korean older adults (≥65 years): defined as “Sufficient” when ≥2000 kcal for males and ≥1600 kcal for females; otherwise, considered insufficient [25].
Omega-3 fatty acid intake	Adequacy of daily omega-3 intake: defined as “Sufficient when ≥1 g; otherwise, considered “Insufficient” [26].
Vitamin D intake	Adequacy of daily vitamin D intake: defined as “Sufficient” when ≥20 µg; otherwise, considered “Insufficient” [25].
Hypertension	A systolic pressure of at least 140 mmHg, a diastolic pressure of at least 90 mmHg, or ongoing treatment with antihypertensive drugs was used to define hypertension [27].
Diabetes	Defined as having a fasting blood glucose level of ≥126 mg/dL, physician-diagnosed diabetes, use of oral hypoglycemic agents or insulin injections, or an HbA1c level of ≥6.5% [28].
Hypercholesterolemia	Defined as a total serum cholesterol level of ≥240 mg/dL or the use of cholesterol-lowering medication [29].
Weight change	Weight change compared to one year ago: “No change” if the weight fluctuation is between 0 and less than 3 kg (increase or decrease), “Weight loss” if the decrease is ≥3 kg, and “Weight gain” if the increase is ≥3 kg.
hs-CRP level	A biomarker for measuring inflammation in the body, analyzed using the particle-enhanced immunoturbidimetric assay with Cobas 8000 (Roche, Munich, Germany). Levels are classified as “<1.0 mg/L” indicating low inflammation risk, and “≥1.0 mg/L” indicating high inflammation risk [30].
Resistance exercise	Categorized as “Yes” if engaging in resistance exercise (e.g., muscle-strengthening activities) at least 2 days per week; otherwise, “No” [31].
Aerobic physical activity	Categorized as “Yes” if engaging in at least 150 min of moderate-intensity physical activity, 75 min of vigorous-intensity physical activity, or a combination of both at equivalent levels per week; otherwise, “No” [31].
Sitting time	Categorized into two groups based on a 10-h threshold, which is known to be associated with an increased risk of cardiovascular disease: those with “10 h or more” and those with “Less than 10 h” [31].
Psychological factors	
Perceived stress level	Categorized as “High” and “Low” based on one’s usual perception of stress in daily life.
Depressive symptom	Categorized as “Yes” if the Korean version of the PHQ-9 [32] score is 10 or higher, or if depressive symptoms have interfered with daily life for two consecutive weeks; otherwise, “No”.
Sleep hour	Categorized as “Less than 7 h”, “between 7 and less than 9 h”, and “9 h or more” [33].
Health perception	One’s usual perception of their own health, categorized as “Good”, “Fair”, or “Poor”.
Body shape perception	One’s usual perception of their own body shape, categorized as “Thin”, “Normal”, or “Obese”.
Social factors	
Area of residence	Classified as “Urban” or “Rural” based on administrative districts.
Household income	The total income earned by a household over one year is classified into five quintiles by Statistics Korea. These quintiles are then reclassified as follows: the upper middle class and high-income groups are categorized as “High”, the middle class as “Middle”, and the lower middle class and low-income groups as “Low”.
Employment status	Categorized as “Yes” if employed, otherwise “No” for unemployed or economically inactive individuals.
Education level	Categorized as “Middle school or below” for those who graduated middle school or lower, and “High school or above” for those who graduated high school or higher.
Living alone status	Categorized as “Living alone” if there are no cohabiting family members or housemates; otherwise, “Not living alone”.
Meal companionship	Categorized as “Eating alone” if meals are mostly taken alone, and “Eating with others” if at least one meal per day is shared with someone else.

Note. SMI, Skeletal muscle index; ASM, Appendicular skeletal muscle mass; PHQ-9, Patient Health Questionnaire-9; hs-CRP, High-sensitivity *C*-reactive protein.

**Table 2:** Characteristics of variables according to body composition abnormalities.

Variables	Categories	Normal (n = 776)	Sarcopenia (n = 392)	Abdominal Obesity (n = 875)	Sarcopenic Obesity (n = 75)
		n * (%) ^†^	n * (%) ^†^	n * (%) ^†^	n * (%) ^†^
Biological factors					
Sex	Male	335 (46.1)	242 (62.5)	367 (43.1)	50 (78.5)
	Female	441 (53.9)	150 (37.5)	508 (56.9)	18 (21.5)
Alcohol consumption	No	475 (59.7)	257 (67.1)	551 (62.2)	49 (65.1)
	Yes	301 (40.3)	135 (32.9)	324 (37.8)	26 (34.9)
Current smoking	No	714 (92.1)	334 (86.0)	800 (91.6)	65 (86.1)
	Yes	62 (7.9)	58 (14.0)	75 (8.4)	10 (13.9)
Protein intake	Insufficient	234 (29.2)	150 (37.9)	294 (32.9)	34 (48.1)
	Sufficient	542 (70.8)	242 (62.1)	581 (67.1)	41 (51.9)
Calcium intake	Insufficient	606 (77.5)	323 (83.9)	709 (80.4)	63 (85.6)
	Sufficient	170 (22.5)	69 (16.1)	166 (19.6)	12 (14.4)
Energy intake	Insufficient	443 (57.7)	295 (77.3)	534 (61.2)	53 (70.8)
	Sufficient	333 (42.3)	97 (22.7)	341 (38.8)	22 (29.2)
Omega-3 fatty acid intake	Insufficient	246 (30.9)	154 (40.0)	297 (33.4)	32 (46.0)
	Sufficient	530 (69.1)	238 (60.0)	578 (66.6)	43 (54.0)
Vitamin D intake	Insufficient	765 (98.7)	389 (99.2)	860 (98.3)	75 (100.0)
	Sufficient	11 (1.3)	3 (0.8)	15 (1.7)	-
Hypertension	No	394 (51.0)	181 (45.0)	242 (27.5)	24 (28.7)
	Yes	382 (49.0)	211 (55.0)	633 (72.5)	51 (71.3)
Diabetes	No	626 (81.0)	311 (79.4)	594 (68.2)	36 (49.6)
	Yes	150 (19.0)	81 (20.6)	281 (31.8)	39 (50.4)
Hypercholesterolemia	No	436 (55.6)	250 (62.1)	434 (51.2)	41 (54.5)
	Yes	340 (44.4)	142 (37.9)	441 (48.8)	34 (45.5)
Weight change	No change	589 (76.9)	292 (73.6)	570 (65.3)	53 (70.7)
	Weight loss	128 (16.1)	78 (21.6)	138 (14.4)	11 (16.1)
	Weight gain	59 (7.0)	22 (4.8)	167 (20.3)	11 (13.2)
hs-CRP level	<1.0 mg/L	594 (76.5)	273 (72.2)	580 (66.2)	43 (57.1)
	≥1.0 mg/L	182 (23.5)	119 (27.8)	295 (33.8)	32 (42.9)
Resistance exercise	No	526 (65.1)	300 (74.8)	679 (77.8)	65 (85.1)
	Yes	250 (34.9)	92 (25.2)	196 (22.2)	10 (14.9)
Aerobic physical activity	No	453 (57.1)	273 (68.8)	601 (67.5)	54 (73.2)
	Yes	323 (42.9)	119 (31.2)	274 (32.5)	21 (26.8)
Sitting time	≥10 h	535 (68.4)	244 (60.8)	530 (60.0)	41 (55.3)
	<10 h	241 (31.6)	148 (39.2)	345 (40.0)	34 (44.7)
Psychological factors					
Perceived stress level	Low	684 (88.5)	340 (87.3)	763 (87.1)	59 (77.9)
	High	92 (11.5)	52 (12.7)	112 (12.9)	16 (22.1)
Depressive symptom	No	719 (93.5)	363 (92.9)	803 (91.7)	68 (91.8)
	Yes	57 (6.5)	29 (7.1)	72 (8.3)	7 (8.2)
Sleep hour	<7 h	382 (48.4)	180 (45.8)	400 (47.0)	25 (36.1)
	7–<9 h	362 (47.7)	184 (46.6)	427 (47.9)	43 (53.9)
	≥9 h	32 (3.9)	28 (7.6)	48 (5.1)	7 (9.9)
Health perception	Good	261 (35.4)	103 (25.9)	235 (28.0)	10 (12.5)
	Fair	355 (44.7)	194 (49.4)	412 (46.2)	33 (44.2)
	Poor	160 (12.0)	95 (24.7)	228 (25.8)	32 (43.2)
Body shape perception	Thin	27 (3.6)	74 (17.2)	3 (0.3)	2 (3.4)
	Normal	747 (96.2)	318 (82.8)	747 (84.6)	71 (94.5)
	Obese	2 (0.2)	-	125 (15.1)	2 (2.1)
Social factors					
Area of residence	Urban	564 (78.8)	269 (74.3)	619 (76.3)	57 (78.5)
	Rural	212 (21.2)	123 (25.7)	256 (23.7)	18 (21.5)
Education level	≤Middle school	438 (52.9)	258 (63.8)	591 (65.6)	49 (66.3)
	≥High school	338 (47.1)	134 (36.2)	284 (34.4)	26 (33.7)
Household income	High	209 (30.7)	67 (19.9)	183 (21.2)	9 (13.6)
	Middle	140 (18.0)	52 (12.4)	157 (19.8)	9 (11.8)
	Low	427 (51.3)	273 (67.6)	535 (59.0)	57 (74.6)
Employment status	No	344 (42.2)	145 (36.5)	385 (43.8)	18 (21.8)
	Yes	432 (57.8)	247 (63.5)	490 (56.2)	57 (78.2)
Living alone status	Living alone	152 (17.4)	96 (22.1)	193 (20.9)	20 (27.8)
	Not living alone	624 (82.6)	296 (77.9)	682 (79.1)	55 (72.2)
Meal companionship	Eating alone	129 (15.1)	99 (24.9)	189 (21.6)	25 (34.3)
	Eating with others	647 (84.9)	293 (75.1)	686 (78.4)	50 (65.7)

Note. * Unweighted; ^†^ Weighted; hs-CRP, High-sensitivity *C*-reactive protein.

**Table 3:** Factors associated with sarcopenia, abdominal obesity, and sarcopenic obesity.

Variables	Categories	Sarcopenia (n = 392)		Abdominal Obesity (n = 875)		Sarcopenic Obesity (n = 75)	
		AOR	95% CI	AOR	95% CI	AOR	95% CI
Biological factors							
Sex (Ref. Female)	Male	3.69	2.64–5.15	1.21	0.87–1.69	7.03	3.48–14.20
Alcohol consumption (Ref. No)	Yes	0.54	0.39–0.74	0.99	0.72–1.35	0.69	0.35–1.39
Current smoking (Ref. No)	Yes	1.32	0.79–2.20	1.07	0.68–1.67	1.01	0.44–2.34
Protein intake (Ref. Sufficient)	Insufficient	0.82	0.61–1.10	1.03	0.74–1.44	1.68	0.84–3.35
Calcium intake (Ref. Sufficient)	Insufficient	0.91	0.63–1.32	1.00	0.75–1.34	1.10	0.55–2.21
Energy intake (Ref. Sufficient)	Insufficient	2.54	1.74–3.70	1.02	0.76–1.37	0.92	0.46–1.85
Omega-3 fatty acid intake (Ref. Sufficient)	Insufficient	1.17	0.79–1.75	0.91	0.66–1.26	1.38	0.77–2.47
Vitamin D intake (Ref. Sufficient)	Insufficient	1.19	0.30–4.75	0.85	0.33–2.20	-	-
Hypertension (Ref. No)	Yes	1.15	0.86–1.54	2.40	1.86–3.09	1.52	0.78–2.96
Diabetes (Ref. No)	Yes	0.93	0.66–1.31	1.72	1.34–2.20	3.25	1.83–5.77
Hypercholesterolemia (Ref. No)	Yes	0.97	0.68–1.38	0.99	0.78–1.27	1.05	0.57–1.94
Weight change (Ref. No change)	Weight loss	1.01	0.69–1.48	1.01	0.73–1.40	0.71	0.29–1.71
	Weight gain	0.80	0.46–1.38	2.81	1.93–4.10	1.73	0.88–3.39
hs-CRP level (Ref. < 1.0 mg/L)	≥1.0 mg/L	1.11	0.79–1.57	1.48	1.10–1.99	2.28	1.36–3.81
Resistance exercise (Ref. Yes)	No	1.68	1.19–2.38	1.57	1.20–2.06	3.15	1.31–7.57
Aerobic physical activity (Ref. Yes)	No	1.26	0.97–1.64	1.26	0.97–1.63	1.42	0.73–2.77
Sitting time (Ref. <10 h)	≥10 h	1.28	0.99–1.66	1.37	1.06–1.76	1.31	0.83–2.08
Psychological factors							
Perceived stress level (Ref. Low)	High	0.99	0.64–1.54	1.05	0.74–1.51	2.55	1.29–5.01
Depressive symptom (Ref. Yes)	No	1.16	0.71–1.89	0.74	0.47–1.19	1.90	0.53–6.81
Sleep hour (Ref. 7–<9 h)	<7 h	0.92	0.70–1.21	0.87	0.70–1.09	0.57	0.32–1.01
	≥9 h	1.43	0.81–2.52	1.18	0.68–2.07	1.44	0.45–4.63
Health perception (Ref. Fair)	Good	0.69	0.50–0.94	0.97	0.71–1.33	0.38	0.19–0.78
	Poor	0.75	0.52–1.08	0.88	0.65–1.19	1.50	0.88–2.55
Body shape perception (Ref. Normal)	Thin	5.72	5.72–5.72	0.10	0.03–0.35	0.71	0.16–3.11
	Obese	-	-	50.99	12.94–200.88	6.47	0.88–47.57
Social factors							
Area of residence (Ref. Urban)	Rural	1.06	0.70–1.60	0.92	0.66–1.28	0.76	0.15–1.68
Education level (Ref. ≥High school)	≤Middle school	1.15	0.87–1.53	1.39	1.08–1.78	0.94	0.46–1.92
Household income (Ref. High)	Middle	1.05	0.61–1.81	1.39	0.91–2.13	1.22	0.37–4.04
	Low	1.66	1.06–2.61	1.27	0.91–1.79	2.08	0.97–4.44
Employment status (Ref. Yes)	No	1.23	0.89–1.72	0.83	0.67–1.05	2.44	1.32–4.53
Living alone status (Ref. Not living alone)	Living alone	0.90	0.59–1.39	1.01	0.70–1.46	0.97	0.46–2.01
Meal companionship (Ref. Eating with others)	Eating alone	2.05	1.36–3.11	1.51	1.03–2.22	3.15	1.21–8.17

Note. AOR, Adjusted odd ratio; 95% CI, 95% Confidence Intervals; Ref., reference; hs-CRP, High-sensitivity *C*-reactive protein.

## Data Availability

The raw data from the 2022 and 2023 KNHANES presented in this study are available at https://knhanes.kdca.go.kr/knhanes/main.do (accessed on 28 February 2025).

## Abbreviations

The following abbreviations are used in this manuscript:

KNHANES	Korea National Health and Nutrition Examination Survey
KDCA	Korea Disease Control and Prevention Agency
SMI	Skeletal muscle index
ASM	Appendicular skeletal muscle mass
PHQ-9	Patient Health Questionnaire-9
hs-CRP	High-sensitivity C-reactive Protein
EPV	Events per variable
AOR	Adjusted odd ratio
95% CI	95% Confidence Intervals
